# The color red attracts attention in an emotional context. An ERP study

**DOI:** 10.3389/fnhum.2015.00212

**Published:** 2015-04-29

**Authors:** Michał Kuniecki, Joanna Pilarczyk, Szymon Wichary

**Affiliations:** ^1^Psychophysiology Laboratory, Institute of Psychology, Jagiellonian UniversityKraków, Poland; ^2^Interdisciplinary Center for Applied Cognitive Studies, University of Social Sciences and HumanitiesWarsaw, Poland

**Keywords:** attention, emotion, visual perception, the color red, event-related potentials

## Abstract

The color red is known to influence psychological functioning, having both negative (e.g., blood, fire, danger), and positive (e.g., sex, food) connotations. The aim of our study was to assess the attentional capture by red-colored images, and to explore the modulatory role of the emotional valence in this process, as postulated by Elliot and Maier (2012) color-in-context theory. Participants completed a dot-probe task with each cue comprising two images of equal valence and arousal, one containing a prominent red object and the other an object of different coloration. Reaction times were measured, as well as the event-related lateralizations of the EEG. Modulation of the lateralized components revealed that the color red captured and later held the attention in both positive and negative conditions, but not in a neutral condition. An overt motor response to the target stimulus was affected mainly by attention lingering over the visual field where the red cue had been flashed. However, a weak influence of the valence could still be detected in reaction times. Therefore, red seems to guide attention, specifically in emotionally-valenced circumstances, indicating that an emotional context can alter color’s impact both on attention and motor behavior.

## Introduction

Color vision aids the visual processing of natural scenes on several levels, such as scene segmentation (Hansen and Gegenfurtner, 2009), object recognition, and stimulus discrimination (Gegenfurtner and Rieger, 2000). Colors may also guide attention towards important objects, since color is used both in nature and culture as a powerful signal (Hutchings, 1997). Information regarding hue is extracted at early stages of visual processing (Gegenfurtner, 2003), which makes its role in triggering early attention shifts plausible. Vivid colors, especially, or those particularly fine-tuned to the perceptual scope of a visual system, can be used to convey messages of two basic kinds: repelling and attracting (King, 2005). In the animal world stripes of contrasting colors, such as seen on wasps, are often used to repel potential predators by signaling unpleasant consequences of attack, however same contrasting colors on a courting bird are an invitation to approach. Interestingly, attraction and repulsion also comprise the basic pleasant-unpleasant dimension of emotion (Lang et al., 1998a), therefore colors may be relevant to the processing of emotional stimuli. In fact, people tend to assign an emotional meaning to particular hues (Moller et al., 2009) and have consistent preferences regarding colors (Palmer and Schloss, 2010). However, some studies reject a general role of color in the processing of emotional stimuli (Bradley et al., 2001; Junghöfer et al., 2001; Codispoti et al., 2011).

Recently, Elliot and Maier (2012) proposed a theory of colors’ impact on psychological functioning. According to their *color-in-context theory*, color carries meaning, and this has a direct and automatic influence on cognitive processes, including attention. This influence is consistent with the emotional evaluation of color as either hospitable or hostile. Thus, color may facilitate approach- or avoidance-oriented psychological processes, serving as an automatically and rapidly processed affective prime. Colors’ connotations stem from pairings of a particular color with experiences, objects and messages, which have sources both in biology and culture. However, these connotations are not uniform, and colors’ impact on behavior is context-dependent. The modulatory role of the context proposed by Elliot and Maier’s (2012) theory is plausible even in case of fast processes like attention switching, given the temporal course of the semantic analysis of visual stimuli. People are able to extract gist of a scene, even when it is presented for only a split second (12–26 ms; Thorpe et al., 1996; Bacon-Macé et al., 2005; Rousselet et al., 2005), and outside of the focus of attention (Li et al., 2002; Peelen et al., 2009). More importantly, emotionally-loaded visual stimuli are promptly discriminated, even when their presentation is very brief (Junghöfer et al., 2001; Schupp et al., 2004), and when they are concealed among distracting neutral stimuli (Öhman et al., 2001; Calvo et al., 2006). The aim of the present study was to test Elliot and Maier’s theory by exploring the engagement of attention by color, and the modulatory role of emotional context in this process.

Red is a particularly good example of the aforementioned properties of colors, which prompted several lines of research within the color-in-context theory framework. Firstly, it was established that viewing red immediately before or during a motor response increases the response’s strength and velocity, most probably due to the elicitation of fear (Elliot and Aarts, 2011). However, when participants are exposed to red several seconds before the motor task, it impairs the motor production, because a relatively distal threat cue causes anxiety rather than fear (Payen et al., 2011). Secondly, red seems to possess emotion-eliciting properties. Moller et al. (2009) have shown that people tend to associate red with negative, danger-bearing emotions, since it is the color of fire, blood, anger, and sometimes of poisonous or dangerous animals. Yet, red does not always signal hostility or danger. Among many species (e.g., primates and fish), red is an evolved biological signal of attractiveness (e.g., Milinski and Bakker, 1990; Waitt et al., 2006). In humans, women and men wearing red clothes are regarded by the opposite sex as more desirable (Elliot et al., 2010; Kayser et al., 2010). The other positive connotation of red is in food, namely in ripe fruits. Notably, detection of ripe fruits is a possible reason why trichromacy has evolved in primates (Sumner and Mollon, 2000; Surridge et al., 2003; Osorio and Vorobyev, 2005). Clearly, red has a double meaning: it may signal either threat or opportunity, depending on the context. This duality is reflected in infants’ preference for red in safe conditions, (i.e., red stimulus primed by a smiling face), and avoidance of this color in threatening circumstances (i.e., red stimulus primed by an angry face; Maier et al., 2009).

The emotional connotation of red switches between negative and positive, but in both emotional extremes red signals the presence of a significant stimulus and thus should require an attentional shift towards it. Indeed, Fortier-Gauthier et al. (2013) have recently shown that simple red targets to be detected among gray distractors evoke larger N2pc than green targets, matched in other physical qualities, pointing to possible special status of the color red in visual search. The N2pc component provides a marker of the spatial location of the attentional focus, being more negative at the posterior scalp locations contralateral to the attended stimulus (Luck and Hillyard, 1994). Before, attentional bias towards red was postulated on a less straightforward basis. Hill and Barton (2005) concluded that wearing red clothes in combat sports significantly increased the chance of victory. Hagemann et al. (2008) argued that this tendency can be explained, at least partially, by the referees’ perceptual bias towards red.

Although attentional bias to red is apparent not only in emotional circumstances (Fortier-Gauthier et al., 2013), the primary context of red seems to be an emotionally arousing one, not one that is calm and neutral. Thus, in line with Elliot and Maier’s (2012) theory, red should have different properties in emotional and neutral circumstances. Provided that signaling is one of the most important functions of color, red should affect attention, particularly in emotional conditions. Indeed, red is the most widespread signaling color in the natural world, as it is easily visible both on the blue background of the sky, and on the green of foliage (Humphrey, 1976). It is also commonly used in the urban environment to raise an alert and draw one’s attention, e.g., through the use of road signs, traffic lights, and significant notices.

We aimed to test alternative accounts of how the color red might influence the processing of emotional visual stimuli. On one side, red might serve as a cue that a given image is important, and thus a shift of attention is desirable. In that case, all red images should capture attention, regardless of their emotional meaning. Alternatively, a possible attentional bias towards red may interact with valence, in which case both positive and negative, but not neutral, red images should attract attention. Finally, red may not influence attention at all, leading to random attending to the red and non-red stimuli.

In order to test the specific influence of red on the capturing of attention, we used a version of the Posner spatial cueing task (Posner et al., 1980), called the “dot-probe”, originally introduced by MacLeod et al. (1986) to study attentional biases towards emotional vs. neutral stimuli. In our modification of this task, a cue comprising a pair of images from the same valence category, one of which always contained a red object while the other did not, were presented simultaneously, one on each side of the screen. They were followed by a target (an asterisk) displayed to the left or right side of the visual field; this target prompted the participant to press the corresponding button on the response box. Comparing reaction times (RTs) in congruent conditions (the target appearing on the side of the red image) to reaction times in incongruent conditions (the target on the side opposite the red image) should allow us to detect an attentional bias towards red in each valence condition. Additionally, we presented cues formed by pairs of images where neither comprised the color red, which created a control condition dubbed “non-aligned”.

Apart from RTs, we also measured the early attentional bias using the event-related potentials (ERP), which can be extracted from the ongoing EEG recording. Changes in event-related potentials may appear faster than any overt behavioral reaction. ERP components that mark shifting of attention in space initiated by cue presentation, particularly early directing attention negativity (EDAN) and subsequent anterior directing attention negativity (ADAN), were of special interest. Both EDAN and ADAN are called event-related lateralizations, as they assume more negative values contralaterally to the location of attentional focus. EDAN exhibits posterior-occipital scalp distribution in the time window between 200 and 400 ms after cue onset. It is hypothesized to represent either a shift of attention caused by decoding the meaning of the cue (Harter et al., 1989; Hopf and Mangun, 2000; Nobre et al., 2000; Talsma et al., 2005; Praamstra and Kourtis, 2010) or the selection of a relevant cue feature (van Velzen and Eimer, 2003; van der Lubbe et al., 2006; Jongen et al., 2007; Brignani et al., 2009). Thereby, according to van Velzen and Eimer (2003), EDAN is a reaction to cue analogous to the target-related N2pc. A following component, ADAN, commencing around 300 ms post cue and lasting till 500 ms has frontal distribution (Praamstra et al., 2005; van der Lubbe et al., 2006) and represents a modality-independent attentional control mechanism (Hopf and Mangun, 2000; Nobre et al., 2000; Eimer et al., 2002, 2003; Seiss et al., 2007, 2009). In addition to the lateralized EEG components, we examined the occipito-parietal P1 component with latency around 100 ms, to check for efficacy of valence manipulation. A number of EEG studies utilizing various image presentation schemes suggest that cortical differentiation between affective categories occurs as early as 100 ms after stimulus onset, evidenced by the modulation of the P1 by valence of presented stimuli (Smith et al., 2003; Carretié et al., 2004; Delplanque et al., 2004; Keil et al., 2007; Van Strien et al., 2009; Bublatzky and Schupp, 2012; Feng et al., 2014; for review, see Olofsson et al., 2008).

According to the interaction hypothesis consistent with Elliot and Maier’s (2012) theory, we expected to observe more prominent EDAN and ADAN components contralateral to the visual field where the red image was shown, especially in the emotional condition. We also anticipated to find shorter reaction times and less errors in trials in which the target appeared in the same visual field as the red image (congruent condition), compared with the incongruent and non-aligned conditions. We assumed that also this effect of the color red would depend on the emotional valence of the stimulus pair, with more pronounced facilitation of the behavioral reactions in case of the emotional stimuli compared with the neutral ones.

## Materials and Methods

### Participants

Twenty-three (5 male and 18 female) students from the Jagiellonian University, Kraków, Poland, with an age range of 18 to 21 years (*M* = 19.3, SD = 0.7), participated in the study for course credit. All participants were right-hand dominant, had normal or corrected-to-normal vision, and did not report any color vision deficiencies. Participants gave written informed consent. The experiment was conducted according to guidelines of the ethic committee of Institute of Psychology, Jagiellonian University in Kraków.

### Stimuli

Forty-five pairs of images from the International Affective Picture System (IAPS; Lang et al., 1999) were selected for the experiment.^1^ The first subset was composed of thirty pairs: ten negative, ten positive, and ten neutral. One image of each pair contained a predominant red object (“Red” in Table 1), the other lacked any shades of red (“Non-red” in Table 1), while both had equal ratings of valence and arousal and, whenever possible, were similar in terms of content and layout. The remaining fifteen pairs (five for each valence category) served as a control condition for the color manipulation (“Non-aligned” in Table 1) with both images in each pair lacking red coloring, but meeting the other criteria of selection described above. Brightness and contrast, calculated as the mean and SD of the luminance component in CIELAB color space, were equated in each pair of images using Adobe Photoshop. Table 1 presents IAPS ratings of images and their basic physical properties compared across experimental conditions. For the sake of clarity in the study, we made sure that the selected pictures depicted neither faces, due to the unique mechanism of face processing (Allison et al., 1999; Alpers et al., 2011; for review, see Dekowska et al., 2008), nor sexual scenes, whose valence and arousal ratings differ largely between men and women (Lang et al., 1999).

**Table 1:** **IAPS ratings (valence and arousal) and basic physical features (luminance, contrast, energy in low and high spatial frequencies) of negative, neutral and positive images, separately for images containing color red, non-red images, and images from control non-aligned condition**.

	Negative			Neutral			Positive			Statistics		
	Red	Non-red	Non-aligned	Red	Non-red	Non-aligned	Red	Non-red	Non-aligned	Valence	Type	Valence × Type
Valence	2.91	2.88	2.80	5.06	5.09	4.97	7.10	7.13	7.17	464,***	0.09, n.s.	0.08, n.s.
Arousal	6.03	5.80	5.92	3.73	3.58	3.37	4.99	4.90	4.95	69,***	0.42, n.s.	0.18, n.s.
Contrast	0.49	0.49	0.55	0.53	0.52	0.52	0.48	0.48	0.50	0.74, n.s.	0.66, n.s.	0.30, n.s.
Luminance	86	87	79	105	106	90	79	78	96	2.8, n.s.	0.03, n.s.	1.1, n.s.
HSF (*z*-score)	−0.20	0.03	−0.32	−0.11	0.04	0.30	−0.31	0.12	−0.19	0.47, n.s.	1.3, n.s.	0.38, n.s.
LSF (*z*-score)	−0.13	−0.06	0.13	0.35	0.44	−0.10	−0.26	−0.38	0.03	2.8, n.s.	0.01, n.s.	1.4, n.s.

Energy in high spatial frequency (HSF; >16 cycles per image) and low spatial frequency (LSF; <8 cycles per image) were calculated using algorithm provided by Delplanque et al. (2007). Luminance and contrast were calculated as the mean and SD of the luminance component in CIELAB color space. ANOVA analyses were performed with factors of valence (negative, positive, neutral) and image type (red, non-red, non-aligned). In statistics columns F_(2,81)_ values for main effects and F_(4,81)_ values for interactions are reported, with ***indicating p < 0.001 and n.s. indicating p > 0.05.

### Procedure

The stimuli were presented against a gray background on a 22-inch flat TFT monitor with a refresh rate of 100 Hz. The software package DMDX (Forster and Forster, 2003) was used to control the experiment. The response pad was connected directly to the recording equipment via an optical transducer.

**Figure 1:**
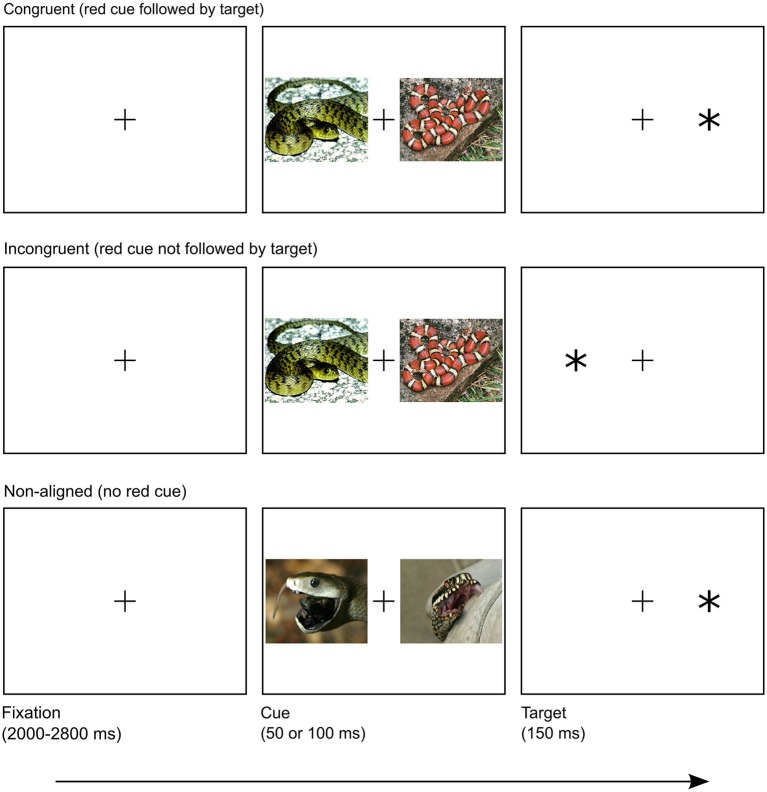
**Overview of trial structure in congruent, incongruent and non-aligned conditions**. Presented images are similar to IAPS images used in the experiment.

The experiment was carried out in a dimly-lit chamber. Participants were seated in a comfortable chair approximately 70 cm away from the screen. After receiving both verbal and written instructions, participants completed the dot-probe task (Figure 1) while the EEG and RTs were recorded. The dot-probe task comprised 360 unique trials (15 pairs × 3 valences × 2 target locations × 2 image locations × 2 SOA), each presented three times in random order, with the restriction that no trials of the same kind were allowed to follow each other. The task was divided into three identical blocks approximately 20 min in length each, with self-paced breaks in between. Before the actual experiment started, participants had the chance to practice the task on ten training trials.

Each trial started with a fixation cross (0.6° × 0.6° in size; positioned in the center of the screen), presented on average for 2400 ms (a random interval of 2000–2800 ms). The fixation cross was followed by a prime: one of the 45 pairs of IAPS pictures, randomly selected and displayed for either 50 or 100 ms, which created two conditions of stimulus onset asynchrony (SOA), fully counterbalanced across the experiment. Two SOA conditions ensured that participants were unable to learn temporal cue-target contingency and hence to prepare the motor response in advance. Relatively short SOA durations are optimal for studying shifts of attention triggered by both emotional (for review see Yiend, 2010) and exogenous factors (Shepherd and Müller, 1989), due to transient influence of both types of cueing on attention deployment. Short SOAs also ensured that the participants did not have enough time to execute a saccade towards either of cues before the target appeared (Calvo et al., 2008). Both images from a pair (each 23.1° wide and 18.3° high) were presented simultaneously, on opposite sides of the screen, spanning from 5.5° to 28.6°, either to the left or the right of the center. Although the pairing of the pictures was permanent, their relative location on the screen was randomized. Worth noting is that all pairs lacking a red object were randomly mixed with pairs in which red was featured. This design served two purposes. Firstly, to maintain uncertainty regarding the color properties of an upcoming cue; this prevented the subjects’ attention from being guided simply by feature-driven pattern. Secondly, to create a control condition in which neither image in a pair contained a red feature, while sharing other properties of experimental pairs, which enabled us to assess the pure role of color. Finally, the target—an asterisk (*, of size 1.2° × 1.2°) appeared for 150 ms, presented 16.7° to the left or right side of the screen. Laterality of the target location was fully counterbalanced across the experiment. The participants were instructed to make a response (as quickly and as accurately as possible) when the asterisk was flashed on the screen, by pressing the button corresponding to the location of the target with their left or right thumb, accordingly. The participants were informed that before each target a pair of images would be flashed and were told not to pay attention to it. No further information about images was given. The next trial immediately followed the response.

Sides of the red image and target display were independent. Combined, they created two conditions: congruent with the target spatially aligned with the red cue, and incongruent with the target and cue appearing on the opposite sides of the screen. Naturally, this division was impossible to maintain in the case of pairs lacking a red feature, due to the absence of the color cue. Hence, those trials formed the non-aligned condition.

### EEG Recording

The EEG was recorded with the ActiveTwo BioSemi system (BioSemi, Amsterdam, The Netherlands) from thirty-two mono-polar locations (Fp1/Fp2, AF3/AF4, F3/F4, F7/F8, FC1/FC2, FC5/FC6, C3/C4, T7/T8, CP1/CP2, CP5/CP6, P3/P4, P7/P8, PO3/PO4, O1/O2, Fz, Cz, Pz, Oz). All the electrodes were placed on the scalp using an Electro-Cap according to the 10–20 system, and referenced to the common mode sense (CMS) electrode with an additional drive right leg (DRL) electrode serving as a ground. The ocular activity was recorded with four bipolar electrodes placed at the outer canthi of each eye for horizontal movements, and above and below the middle of the left eye for vertical movements. An additional two electrodes were placed at both mastoids. The EEG signal recorded at a sampling rate of 512 Hz with a 104 Hz anti-aliasing filter was continuously stored on a computer for offline analysis. Using BrainVision analyzer software (BrainProducts GmbH), the data were offline re-referenced to the average of the mastoids, and filtered with band pass filter 0.016–30 Hz (12 dB). Subsequently, segments of 600 ms in duration were extracted for each trial, starting 100 ms before the image onset. The first 100 ms period of each segment was defined as a baseline. Data were edited for the artifacts by rejecting trials with zero activity, voltage steps over 50 μV, and voltage differences larger than 100 μV. Segments contaminated by ocular artifacts were excluded from the analysis by rejecting trials with HEOG activity greater than 40 μV. Artifact-free trials in which participants correctly responded to the target stimulus, were averaged separately for valence type (positive, neutral, negative) location of red cue (left and right visual field), and SOA (50 or 100 ms). On average 55.4 trials (SD = 4.9) were used to calculate ERP waveforms, after rejection of 7.5% of trials (Table 2). ANOVA analysis conducted on the number of averaged trials with factors of valence, location of the red cue, and SOA revealed no significant main effects. Only the interaction of valence with location of cue proved to be significant *F*_(2,42)_ = 5.3, *p* = 0.009. Pairwise comparison revealed that this interaction was due to difference in positive condition. It is unlikely that this effect impacted on our ERP results, since there was no corresponding effect in either EDAN (*F*_(2,42)_ = 0.84, *p* = 0.44), or ADAN (*F*_(2,42)_ = 0.19, *p* = 0.83) components.

**Table 2:** **Mean number of artifact-free trials (with SD reported in brackets) used to calculate EDAN and ADAN components for all valence (negative, neutral, positive), SOA (50 ms and 100 ms), and location of red cue (left or right visual field) conditions**.

	SOA 50 ms		SOA 100 ms	
	Left VF	Right VF	Left VF	Right VF
Negative	56 (4.2)	55.4 (5.4)	55.3 (5.2)	55.9 (5.2)
Neutral	55.1 (4.7)	55.1 (5.5)	55.9 (5.2)	55.4 (5.3)
Positive	54.7 (5.1)	55.5 (5.5)	54.5 (5.3)	56.5 (4.1)

Lateralized EDAN and ADAN potentials were calculated using standard procedure involving averaging of mean EEG activity recorded contralaterally and ipsilateraly in respect to the location of the red cue (Seiss et al., 2009). Since for every cue in any valence and SOA conditions there was the same number of targets in the left and right visual fields, subtraction and averaging procedure over left and right electrode locations eliminated the overlap of cue and target-related EEG activity, including motor preparation (for discussion of this issue see Luck, 2014; for similar experimental design see Ansorge et al., 2009). Following Praamstra et al. (2005, 2009), we scored EDAN and ADAN as a mean EEG activity over pooled electrode locations. The specific time windows and electrode locations were determined using grand-average waveforms, and scalp topographies. For EDAN, with time window 200–250 ms, we averaged electrode pairs O1/O2, P3/P4, PO3/PO4, and P7/P8, while for ADAN, with time window 300–350 ms, the following ones: FC1/FC2, AF3/AF4, F3/F4, and F7/F8. Non-aligned trials were not taken into account in this analysis, as they lacked a laterally-located cue which could serve as a reference.

The non-lateralized P1 component in response to cue onset was scored as a mean EEG activity on parietal and occipital locations (PO4, O2, Oz, O1, PO3) in time window 70–100 ms. The P1 was calculated for each valence condition, regardless whether a pair was congruent, incongruent or non-aligned.

### Statistical Analyses

A valence (positive, neutral, negative) by congruency (congruent, incongruent, non-aligned) repeated measures ANOVA was conducted on reaction times (medians aggregated for participant and condition) and arc-sine transformed mean error rates. Reaction time analysis was performed on all correct responses between 100 and 1000 ms.

The mean amplitude of P1 component was analyzed using repeated measures ANOVA with factors of valence (positive, neutral, negative), electrode (PO4, O2, Oz, O1, PO3) and SOA (50 and 100 ms). With respect to EDAN and ADAN components two complementary analyses were conducted. Firstly, mean raw ERP data over pooled locations in EDAN and ADAN time windows were investigated using repeated measures ANOVA with factors of valence (positive, neutral, negative), red cue visual field (left, right) lateralization of electrode cluster (ipsilateral, contralateral to the red cue) and SOA (50 and 100 ms). Secondly, lateralized EDAN and ADAN components were analyzed using repeated measures ANOVA with factors of valence (positive, neutral, negative), and SOA (50 and 100 ms). In all ANOVA analyses with factors featuring more than two levels, if the sphericity assumption was violated, the Huynh-Feldt correction was applied, and the adjusted *p*-values are reported. All simple effects were investigated using *post hoc* comparisons with the Bonferroni correction. Data from one participant had to be rejected from the analyses due to very high error rates (29.5% vs. the average of 7.5%), leaving data from 22 participants for all further analyses.

## Results

### Behavioral Performance

#### Accuracy

**Figure 2:**
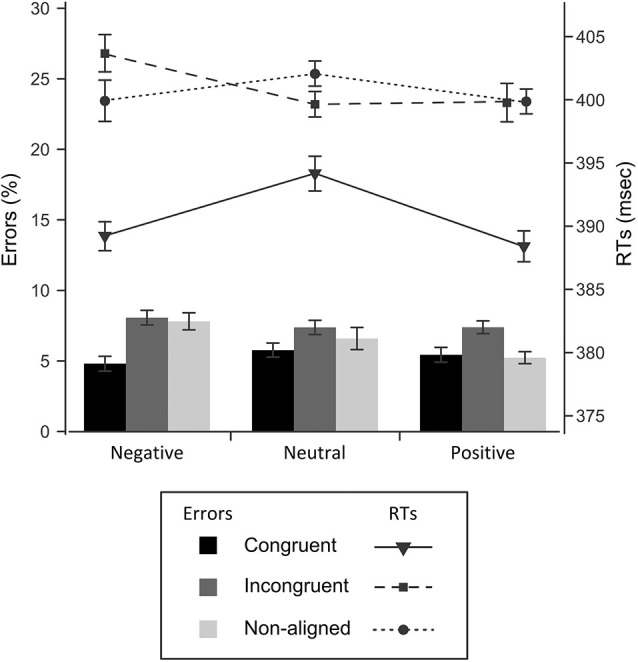
**Response times (line graphs) and error rates (bar graphs) for each experimental condition**. Standard errors were corrected for a repeated measures analysis (Cousineau, 2005).

The overall pattern of accuracies and reaction times with respect to congruency and valence is shown in Figure 2. Error rates were affected by congruency (*F*_(2,42)_ = 6.9, *p* = 0.002, $\eta_{p}^{2}$ = 0.248), such that participants’ responses were most frequently correct in the congruent (94.7%), and least correct in the incongruent condition (92.4%), with intermediate accuracy in the non-aligned condition (93.5%). It seems that, as expected, a red cue facilitated responding on the side where it appeared, causing both a decrease of performance in the incongruent condition, and its increase in the congruent condition, whereas absence of the color cue indeed acted as a neutral baseline. Also, the valence altered response accuracy (*F*_(2,42)_ = 7.0, *p* = 0.002, $\eta_{p}^{2}$ = 0.251). That is, participants performed slightly more accurately in the positive (93.9%) than in the neutral condition (93.4%), and the lowest accuracy was observed in the negative condition (93.1%). Most importantly, valence and congruency factors produced an interaction effect (*F*_(4,84)_ = 3.7, *p* = 0.008, $\eta_{p}^{2}$ = 0.149), which was further investigated using simple effects tests. In the negative valence condition, congruency resulted in significantly higher accuracy than incongruency (*p* = 0.006), or the absence of the red cue (*p* = 0.002). The positive primes elicited a slightly distinct pattern of response: participants performed the task more correctly both in the congruent and non-aligned trials, compared with the incongruent trials (*p* = 0.033 and *p* = 0.019, respectively). Finally, the analysis revealed that in the neutral condition, congruency of the prime did not alter accuracy significantly.

#### Reaction Times Time-Locked to Target

Overall, participants were faster to respond when the target was preceded by the red cue (*M* = 392 ms), than in the incongruent (402 ms) or the non-aligned condition (402 ms), which was reflected in the effect of congruency (*F*_(2,42)_ = 39.7, *p* < 0.001, $\eta_{p}^{2}$ = 654). Although there was no significant main effect of valence, the emotional content of the cue interacted with the congruency factor, affecting the reaction times significantly (*F*_(4,84)_ = 3.8, *p* < 0.007, $\eta_{p}^{2}$ = 154). A follow-up analysis revealed that in the congruent condition both negative and positive cues shortened the response time, in comparison to the neutral cue condition, by 4.9 ms (*p* = 0.036) and 7.8 ms (*p* = 0.01), respectively. On the contrary, an incongruent cue or the absence of red cue evoked no differences in reaction times with respect to valence. Summing up, this pattern of results indicates that red is an effective cue on the behavioral level, reducing the latency of reaction to the target, whereas the overall emotional charge of a given pair of images bears only an indirect relationship to RTs, enhancing the effect of color cueing.

### Cue-Locked ERP Components

**Figure 3:**
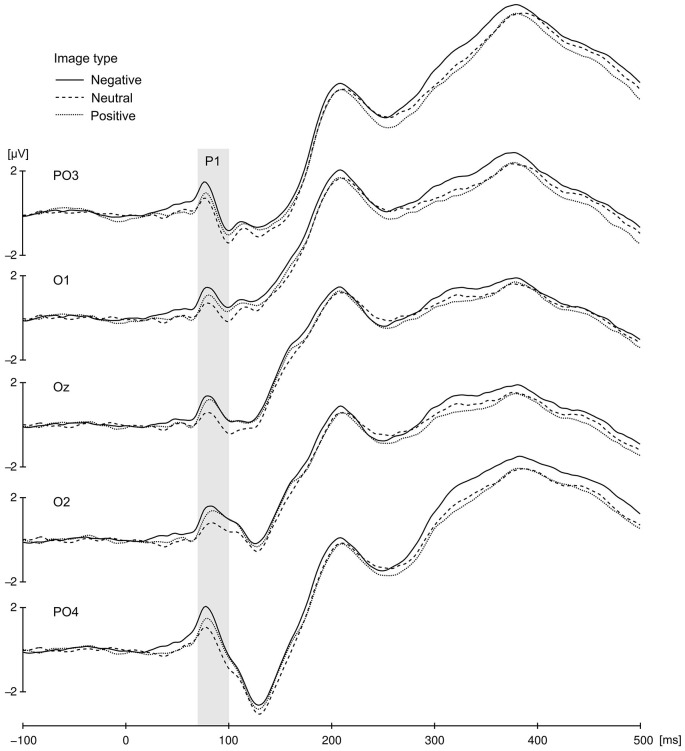
**Cue-locked P1 component for negative, neutral and positive condition elicited at occipito-parietal locations**. The presented data were averaged across all congruency and SOA conditions. Gray bar represent P1 time window.

The P1 component elicited at occipito-parietal locations proved to be sensitive to the affective value of presented images (Figure 3). Differences in the P1 amplitude evoked by negative (*M* = 1.1, SD = 3.1), positive (*M* = 0.66, SD = 2.9), and neutral cues (*M* = 0.33, SD = 2.81) were significant, as validated by main effect of valence, *F*_(2,42)_ = 10.13, *p* < 0.001, $\eta_{p}^{2}$ = 0.325. Pairwise comparison revealed that P1 elicited by negative cues differed significantly from P1 elicited by neutral (*p* = 0.004) and postive cues (*p* = 0.021), but P1 to positive cues did not differ from P1 to neutral ones (*p* = 0.14). No other effects or interactions were significant in the P1 amplitude analysis.

**Figure 4:**
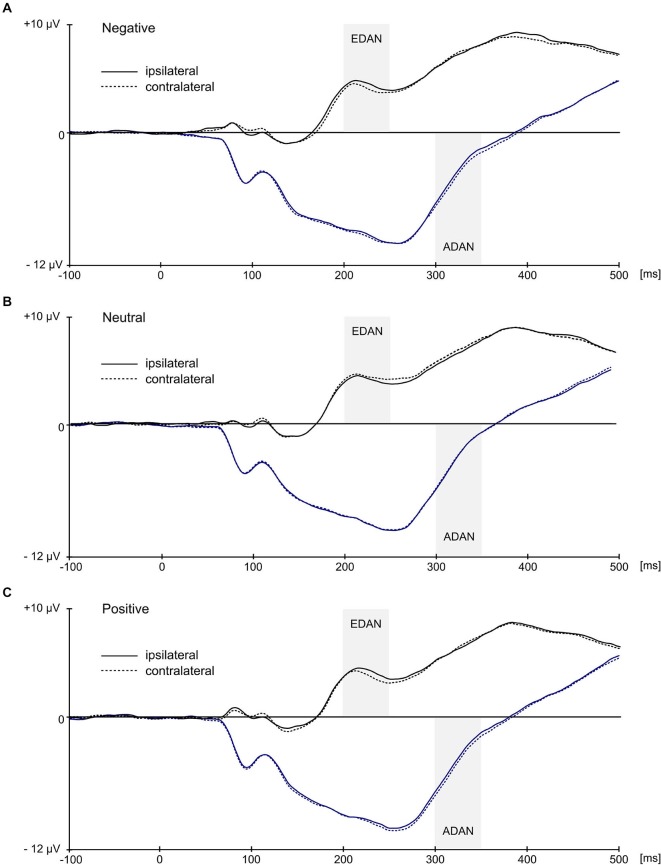
**Grand average ERPs ipsilateral and contralateral to the visual field of the red image**. Black waveforms illustrate early directing attention negativity (EDAN) component and were averaged across occipito-parietal (O1/O2, P3/P4, PO3/PO4, P7/P8) locations. Blue waveforms illustrate anterior directing attention negativity (ADAN) component and were averaged over frontal locations (FC1/FC2, AF3/AF4, F3/F4, F7/F8). Gray bars represent time-windows for EDAN and ADAN.

Statistical analysis confirmed the presence of EDAN component (Figure 4) by significant effect of lateralization (*F*_(1,21)_ = 6.7, *p* = 0.017, $\eta_{p}^{2}$ = 0.242) with potentials assuming smaller values at electrodes contralateral to the red cue (*M* = 3.97, SD = 2.7) than at electrodes ipsilateral to the red cue (*M* = 4.13, SD = 2.7). Crucially, substantiating the influence of context on attention deployment, the interaction between valence and laterality proved to be significant, *F*_(2,42)_ = 10.5, *p* < 0.001, $\eta_{p}^{2}$ = 0.333. Further investigation of this interaction with simple effects revealed that in the negative and positive conditions values at contralateral electrodes were more negative (*M* = 3.98, SD = 2.75 and *M* = 3.68, SD = 2.72, respectively) than at ipsilateral electrodes (*M* = 4.33, SD = 2.86 and *M* = 4.03, SD = 2.73, respectively), while in the neutral condition an opposite effect emerged with potentials at contralateral electrodes being slightly more positive (*M* = 4.25, SD = 2.79) than at ipsilateral ones (*M* = 4.02, SD = 2.63). These simple effects were statistically significant, with *p* = 0.005 for negative, *p* = 0.001 for positive, and *p* = 0.045 for neutral condition. Furthermore, the main effect of SOA proved to be significant (*F*_(1,21)_ = 8.94, *p* = 0.007, $\eta_{p}^{2}$ = 0.299), as well as interaction of SOA with red cue visual field (*F*_(1,21)_ = 4.71, *p* = 0.042; $\eta_{p}^{2}$ = 0.183). This effect was driven by the overall larger values of mean EEG activity in 50 ms SOA condition as compared with 100 ms SOA condition, especially if the red cue appeared in the left visual field (*M* = 4.55, SD = 3.77 for 50 ms SOA and *M* = 3.51, SD = 3.72 for 100 ms SOA condition), as compared with right visual field (*M* = 4.31, SD = 2.5 for 50 ms SOA and *M* = 3.82, SD = 2.26 for 100 ms SOA condition).

**Figure 5:**
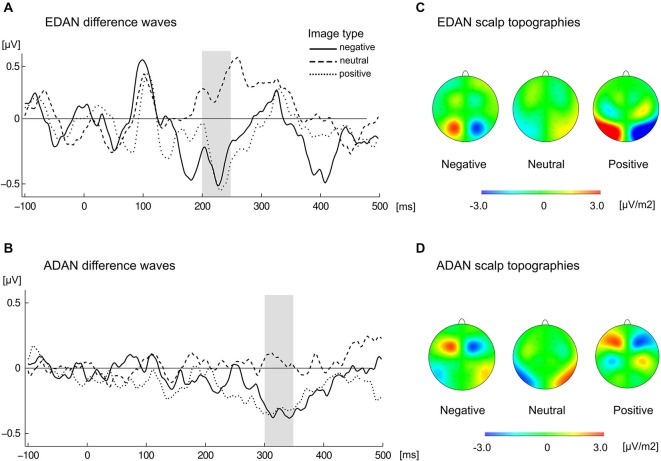
**Cue-locked grand average waveforms for (A) EDAN and (B) ADAN, obtained by subtracting ERPs at ipsilateral from contralateral locations to the red image, separately for negative, neutral, and positive slides**. EDAN was calculated from pooled parietal locations (O1/O2, P3/P4, PO3/PO4, P7/P8) and ADAN from pooled frontal locations (FC1/FC2, AF3/AF4, F3/F4, F7/F8). The gray bar indicates time-window used to obtain amplitudes for corresponding effects. Scalp topographies present difference in activity contra-ipsilateral to the red cue for the left hemisphere and difference ipsi-contralateral for the right hemisphere in the time window of EDAN **(C)** and ADAN **(D)** components.

Calculation of lateralized EDAN produced an enhanced parietal negativity on the scalp contralateral to the red cue in both emotional conditions, while in the neutral condition slightly opposite effect emerged (Figure 5). This resulted with EDAN assuming negative values in both positive (*M* = −0.35, SD = 0.43) and negative valences (*M* = −0.34, SD = 0.51), as opposed to neutral one (*M* = 0.23, SD = 0.51). The difference was overall significant (*F*_(2,42)_ = 10.5, *p* < 0.001, $\eta_{p}^{2}$ = 0.333), and pairwise comparisons revealed that it was indeed driven by neutral condition differing significantly from both negative (*p* < 0.004) and positive (*p* < 0.001) conditions. Neither main effect of SOA nor its interaction with valence reached statistical significance.

Similarly, the ADAN component (Figure 4) was substantiated by significant main effect of lateralization (*F*_(1,21)_ = 13.4, *p* = 0.001, $\eta_{p}^{2}$ = 0.39), which confirmed that potentials at contralateral electrodes were more negative (*M* = −3.73, SD = 4.02) than at ipsilateral electrodes (*M* = −3.53, SD = 4.11). Again, significant interaction of lateralization with valence (*F*_(2,42)_ = 5.72, *p* = 0.006, $\eta_{p}^{2}$ = 0.214) supported the idea of modulatory influence of context on attentional shift. A follow up analysis of simple effects showed that ADAN was more negative at contralateral sites than at ipsilateral sites for negative cues (*M* = −3.74, SD = 4.19 for contralateral and *M* = −3.39, SD = 4.3 for ipsilateral location) and for positive cues (*M* = −3.93, SD = 4.02 for contralateral and *M* = −3.6, SD = 4.07 for ipsilateral location), while in case of neutral cues this difference was negligible (*M* = −3.53, SD = 4.05 for contralateral and *M* = −3.6, SD = 4.14 for ipsilateral locations). Consequently, simple effect of laterality was significant in case of negative (*p* = 0.003) and positive conditions (*p* = 0.004) but not in case of neutral condition (*p* = 0.46). Additionally, main effect of SOA was also significant (*F*_(1,21)_ = 59.35, *p* < 0.001, $\eta_{p}^{2}$ = 0.739) with smaller mean EEG activity in 50 ms SOA condition (*M* = −2.48, SD = 3.87) as compared with 100 ms SOA condition (*M* = −4.78, SD = 4.34).

Lateralized ADAN (Figure 5) exhibited negative deflection in positive (*M* = −0.33, SD = 0.47) and negative (*M* = −0, 35, SD = 0.48) conditions as compared to neutral one (*M* = 0.06, SD = 0.41). This significant effect (*F*_(2,42)_ = 5.72, *p* < 0.006, $\eta_{p}^{2}$ = 0.214) was driven by the difference between negative and neutral (*p* < 0.026) and positive and neutral (*p* < 0.038) conditions. Neither main effect of SOA nor its interaction with valence proved significant.

In summary, the attention-capturing properties of the color red seemed to be modulated by the overall emotional value of stimuli. When presented images were emotionally charged, the EDAN and ADAN components reflected an attention shift towards the red image. On the other hand, in case of the neutral pairs, the data indicated no significant shift of attention.

## Discussion

In the present study, using a dot-probe task coupled with EEG recording, we demonstrated that the color red captures attention and facilitates congruent motor response, particularly in an emotional context.

Participants were faster and more accurate in their responses when the target followed a red-colored cue. The effect was significantly stronger when the color cue was emotionally loaded, revealing interaction between the physical feature and the emotional valence of the image. This is consistent with propositions put forward by Elliot and Maier (2012) that color influences psychological functioning, and that the actual manifestation of this influence depends on the context. Specifically, it appears that the color red generally facilitates motor output. The magnitude of this effect is potentiated by the emotional context, a phenomenon most likely mediated by the evolutionary meaning of red, which serves as a significant signal, both in the appetitive and the aversive conditions.

The hypothesis postulating an early automatic shift of attention by red emotional images is corroborated by the analysis of the event-related potentials. We found that both the EDAN and ADAN components were larger contralateral to the red cue, but only if the cue was emotional. We also observed significant effects of SOA on raw EEG in both EDAN and ADAN time windows. However, these effects do not interact with laterality, representing EEG activity averaged across contra- and ipsilateral electrode locations. As we are only interested in comparing activity contra- and ipsilateral to the red cue, our main experimental effect is the interaction of laterality with valence. From this perspective effects of SOA that do not interact with laterality cannot be interpreted in any meaningful way. Moreover, SOA effects disappear completely when analysis is conducted on the subtracted data.

Cueing by color observed only after emotional images suggests that the appearance of an emotional image increased the visual cortex activation linked to the processing of a potentially significant stimulus. This in turn might have led to enhanced cortical susceptibility to the evolutionarily relevant features, such as red coloration. Indeed, the visual cortex activation, evidenced by the magnitude of the P1 component, was modulated by valence of the presented cue within the first 100 ms after image onset. The P1 evoked by both negative and positive cues was numerically larger than evoked by neutral cues. However, it was most eminent in the negative condition and proved to differ significantly from the P1 in positive and neutral conditions, possibly reflecting so-called negativity bias (Smith et al., 2003; Delplanque et al., 2004; Bublatzky and Schupp, 2012; Feng et al., 2014). It has been suggested that the P1 enhancement in emotional context reflects more efficient sensory processing of potentially relevant stimuli (Smith et al., 2003; Carretié et al., 2004; Keil et al., 2007; Bublatzky and Schupp, 2012; Pourtois et al., 2013; Feng et al., 2014). The evidence indicating that emotional stimuli boost the activation of visual cortex has been also obtained in neuroimaging studies (e.g., Lang et al., 1998b; Kuniecki et al., 2003).

Closer examination of color with valence interaction reveals that in contrast to the emotional conditions, in the neutral condition the EDAN component proved to be more pronounced ipsilateral to the red image. This result might appear somewhat surprising, since it suggests that in the case of neutral stimuli, early attention was directed away from the red cue. Observed lack of attention engagement in neutral condition might be explained by the superfluous nature of the color red in a non-emotional context, due to a lack of evolutionary significance in the neutral images. Therefore, in the case of the neutral stimuli, attention was either not as focused as it was in the case of the emotional stimuli, or it was quickly directed away from the red image.

Reaction times support this hypothesis, as the difference in RTs between congruent and incongruent trials was significantly larger in the negative and the positive, compared with the neutral condition. The occurrence of a red image caused the shift of attention chiefly in the emotional conditions, which must have affected motor preparation accordingly. Indeed, shifts of the exogenous attention are in most cases linked to motor preparation (Sheliga et al., 1997). Uncoupling of those two processes is possible, though it requires very specific experimental manipulation (Praamstra and Oostenveld, 2003; Praamstra et al., 2005, 2009; Cespón et al., 2012). Elliot and Aarts (2011) obtained evidence that the occurrence of the color red, as compared with blue and gray, enhanced motor processes, such as the time and strength of a reaction. Facilitated motor responses corresponding to attentional preference for red over green targets were observed also in Fortier-Gauthier et al. (2013) study. The authors concluded that this property of red, observed also in our study, should be taken into account while choosing stimuli for an experiment with reaction times’ measurement.

Although the EDAN stands for early directing attention negativity it is by no means “early” in terms of the chronometry of visual processing. Foxe and Simpson (2002) have shown that evoked potentials occurring around 200 ms after a stimulus onset do not represent the first feedforward sweep of visual information reaching the visual cortex. Instead, they arise as a result of multiple iterations of interactive processing between several cortical and possibly subcortical areas. Accordingly, the observed modulation of the P1 by valence substantiates the idea that differences in the following EDAN component represented the combined influence of emotional valence and coloration, because the visual analysis of both of these features must have been considerably advanced at this stage.

Indeed, the influence of both semantic and physical features on the EDAN component was observed in earlier studies. Presenting an asymmetric cue, such as an arrow, can affect EDAN due to difference in physical features rather than due to the meaning of the cue. van Velzen and Eimer (2003) showed that the EDAN component is not dependent on the direction the cue is pointing to, but instead on the spatial asymmetry of the cue itself. Later it was confirmed that EDAN is related to the asymmetry inherently present in arrow cues (Jongen et al., 2007; Brignani et al., 2009). Additionally, pairs of symmetric cues do not evoke EDAN, even if they are valid, as shown by Brignani et al. (2009) who cued target location by identical textures. On the other hand, Ranzini et al. (2009) found that in certain conditions EDAN can be elicited by completely symmetrical cues, namely by centrally presented numbers. They explain this result in terms of “spatial-numerical association of response codes” (SNARC) theory, stipulating overlearned automatic association between numerals and location in space (Dehaene et al., 1993), i.e., large numbers are mentally associated with the right part of the space, whereas small numbers are associated with the left part (Hubbard et al., 2005). The numerals were effective cues, shifting attention and influencing EDAN, therefore their meaning must have been decoded before the target commenced. In our experiment, the cue images were identical in terms of emotional valence and basic visual properties. The asymmetry was created by the presence of the red hue. Interestingly, the color asymmetry on its own did not influence EDAN in neutral condition, but only did so in emotional condition. Therefore, it seems that in our case the effect of physical asymmetry was modulated by rapid decoding of the images’ semantic meaning. It is plausible that this interaction stems from overlearned association between red hue and emotional stimuli resembling in that aspect the SNARC effect.

Additionally, because cues provided by the IAPS slides were irrelevant to the task, we assume that the enhanced EDAN component reflected a perceptual and automatic bottom-up process, rather than a more elaborate, decision-based top-down process. It appears that even if the explicit instruction requires the participant to ignore the color cue, the early detection system working in the background is unable to completely disregard the color as a valuable hint. This supports Elliot and Maier’s idea that color influences psychological functioning automatically. Indeed, Theeuwes (1992), and Kim and Cave (1999), have shown that an irrelevant-to-the-task but salient color singleton captures spatial attention. This effect persists even if participants are informed in advance that the colored singleton should be ignored (Theeuwes et al., 2006). Importantly, these effects do not depend on any special quality of the color, but rather on its perceptual distance to its surroundings. This might raise doubts if in our case the observed effect is also color-unspecific, and relies chiefly on the relative consistency of the red hues dominating over the background. Although it is not feasible to entirely refute this possibility given our experimental design, the significant interaction of EDAN and ADAN with the valence of images provides strong support for the idea that the color red was not determined to be attention-worthy throughout all stimulus presentations, but only in those with emotional content. Thus, the effect we report here cannot be exhaustively explained by a simple color pop-out. We are not able to conclude if this effect is exclusive for red hue, as we do not contrast effects of red and a single different color. It is possible that other hue would also produce similar results, even regardless of its emotional connotations. However, the unique influence of red on facilitation of motor responses and attentional preference was observed in studies directly comparing effects of different hues (Elliot and Aarts, 2011; Payen et al., 2011; Fortier-Gauthier et al., 2013).

In our experiment the target immediately followed the cue, which might lead to the conclusion that effects attributed to shifts of attention are in fact related solely to motor preparation. Our results however do not provide support for such interpretation. Firstly, the occipito-parietal topography of EDAN and frontal topography of ADAN components are in agreement with literature (Hopf and Mangun, 2000; Nobre et al., 2000; Praamstra et al., 2005; Praamstra and Kourtis, 2010; Van der Lubbe and Utzerath, 2013). Importantly, neither of these components exhibited central-frontal distribution characteristic to motor activity. Secondly, calculation of EDAN and ADAN involved averaging over the same number of left and right target occurrences, therefore cancelling out any attention-unrelated (i.e., motor) lateralizations (Luck, 2014). Double subtraction procedure used for the calculation of EDAN and ADAN, as well as N2pc, compensates also for the overlap of EEG activity evoked by cue and target, leaving only cue-locked activity (Ansorge et al., 2009). It is important to note, however, that ADAN can be evoked not only by attentional shift, but also by motor preparation (Praamstra et al., 2005, 2009; Eimer and van Velzen, 2006; van der Lubbe et al., 2006; Gherri et al., 2007). Motor-related ADAN is bound to the presentation of the cue predicting movement direction or hand selection. The more unambiguous the cue in respect to the expected motor activity, the larger the ADAN component (Praamstra et al., 2009). In our case the cues were irrelevant to the location of the forthcomming target and hence were not valid. It is therefore unlikely that the cue-locked differences observed in the ADAN time window are entirely related to motor processes.

Lastly, it is worth noting that relatively few studies have explicitly tested how emotional processing impacts visual perception. For example, Brosch et al. (2011) explored the relative influence of task-irrelevant emotional cues (angry faces), and exogenous cues (a bright border placed around an image) on early attention deployment indexed by the N2pc component. They established that while the exogenous, physical cue influenced the N2pc, the valence of the cue did not. While this result is inconsistent with the majority of findings exploring the engagement of attention by emotional faces (clearly showing modulation of the N2pc by emotional cues; Eimer and Kiss, 2007; Fenker et al., 2010; Feldmann-Wüstefeld et al., 2011; Ikeda et al., 2013), it provides evidence that—under specific experimental conditions—the exogenous cueing in the emotional dot-probe paradigm is capable of capturing attention. Our results are analogous, insofar as red coloration of the image is regarded as a kind of exogenous cue. However, the present study suggests that the emotionality of the cue plays a role too, by modulating the attention capture through the evolutionarily relevant physical feature.

Phelps et al. (2006), in a purely behavioral study using facial expressions as emotional cues, showed that emotion interacts with early visual processing by lowering the contrast required to identify target stimuli. Working in the same vein, Bocanegra and Zeelenberg (2009) obtained evidence that emotion indeed improves discrimination, but only of stimuli with low spatial frequency. Conversely, detection of high spatial frequency stimuli was hindered by the prior exposition of emotional faces. This finding is corroborated by evoked potential evidence showing that low, but not high, spatial frequencies are crucial for early discrimination of emotional from neutral stimuli (Alorda et al., 2007; Carretié et al., 2007). These results suggest that the emotional context selectively facilitates perception of specifically emotion-related aspects of a visual stimulus. Since the color red possesses a signaling value characteristic for the emotional content (e.g., Humphrey, 1976; Elliot and Pazda, 2012), our findings provide further evidence which strengthens this hypothesis.

In summary, our results fit into the color in context theory advocated by Elliot and Maier (2012), as the impact of the color red on motor behavior and attentional shifts is modulated by the emotional context of the presented stimuli. At the relatively early stage of visual processing, only the emotional red stimuli attract attention, while the neutral red images are ignored. At the behavioral level, the color red speeds response in all conditions, however the magnitude of this gain is context-dependent. Furthermore, this process seems to be purely automatic and bottom-up in nature. Our results suggest also that the color of stimuli should be controlled in studies concerning reaction times and attention, especially if the presented stimuli have emotional significance.

## Conflict of Interest Statement

The authors declare that the research was conducted in the absence of any commercial or financial relationships that could be construed as a potential conflict of interest.

## Footnotes

^1^Experimental pairs: 9405-6350, 3140-9301, 9253-9140, 8485-9050, 9630-9280, 9622-9600, 9373-1275, 8480-9490, 3250-1932, 1051-1070 (negative), 7352-1942, 7282-7481, 8371-8116, 8340-8193, 8090-8500, 1604-5891, 8510-5836, 5480-5600, 7330-1610, 5830-5833 (positive), 9635.2-9913, 2690-2446, 7184-7055, 7185-7059, 7056-6150, 1616-1675, 7058-7057, 5520-5530, 5395-7546, 7820-7188 (neutral). In each experimental pair, the first number indicates the red image. Control pairs: 9901-9903, 6510-6570.1, 9290-9330, 6200-6210, 1052-1050 (negative), 8341-8179, 1740-1510, 7480-5820, 5820-5814, 1440-1460 (positive), 7040-7030, 7038-5534, 7484-7487, 7100-5740, 7247-7249 (neutral).

## References

[B1] Allison T. Puce A. Spencer D. D. McCarthy G. (1999). Electrophysiological studies of human face perception. I: potentials generated in occipitotemporal cortex by face and non-face stimuli. Cereb. Cortex 9, 415–430. 10.1093/cercor/9.5.415 10450888

[B2] Alorda C. Serrano-Pedraza I. Campos-Bueno J. J. Sierra-Vázquez V. Montoya P. (2007). Low spatial frequency filtering modulates early brain processing of affective complex pictures. Neuropsychologia 45, 3223–3233. 10.1016/j.neuropsychologia.2007.06.017 17681356

[B3] Alpers G. W. Adolph D. Pauli P. (2011). Emotional scenes and facial expressions elicit different psychophysiological responses. Int. J. Psychophysiol. 80, 173–181. 10.1016/j.ijpsycho.2011.01.010 21277913

[B4] Ansorge U. Kiss M. Eimer M. (2009). Goal-driven attentional capture by invisible colors: evidence from event-related potentials. Psychon Bull. Rev. 16, 648–653. 10.3758/pbr.16.4.648 19648447 PMC2780736

[B6] Bacon-Macé N. Macé M. J. Fabre-Thorpe M. Thorpe S. J. (2005). The time course of visual processing: backward masking and natural scene categorisation. Vision Res. 45, 1459–1469. 10.1016/j.visres.2005.01.004 15743615

[B5] Bocanegra B. R. Zeelenberg R. (2009). Emotion improves and impairs early vision. Psychol. Sci. 20, 707–713. 10.1111/j.1467-9280.2009.02354.x 19422624

[B7] Bradley M. M. Codispoti M. Cuthbert B. N. Lang P. J. (2001). Emotion and motivation I: defensive and appetitive reactions in picture processing. Emotion 1, 276–298. 10.1037//1528-3542.1.3.276 12934687

[B8] Brignani D. Guzzon D. Marzi C. A. Miniussi C. (2009). Attentional orienting induced by arrows and eye-gaze compared with an endogenous cue. Neuropsychologia 47, 370–381. 10.1016/j.neuropsychologia.2008.09.011 18926835

[B9] Brosch T. Pourtois G. Sander D. Vuilleumier P. (2011). Additive effects of emotional, endogenous and exogenous attention: behavioral and electrophysiological evidence. Neuropsychologia 49, 1779–1787. 10.1016/j.neuropsychologia.2011.02.056 21382388

[B10] Bublatzky F. Schupp H. T. (2012). Pictures cueing threat: brain dynamics in viewing explicitly instructed danger cues. Soc. Cogn. Affect. Neurosci. 7, 611–622. 10.1093/scan/nsr032 21719425 PMC3427861

[B11] Calvo M. G. Avero P. Lundqvist D. (2006). Facilitated detection of angry faces: initial orienting and processing efficiency. Cogn. Emot. 20, 785–811 10.1080/02699930500465224

[B12] Calvo M. G. Nummenmaa L. Hyönä J. (2008). Emotional scenes in peripheral vision: selective orienting and gist processing, but not content identification. Emotion 8, 68–80. 10.1037/1528-3542.8.1.68 18266517

[B13] Carretié L. Hinojosa J. A. López-Martín S. Tapia M. (2007). An electrophysiological study on the interaction between emotional content and spatial frequency of visual stimuli. Neuropsychologia 45, 1187–1195. 10.1016/j.neuropsychologia.2006.10.013 17118408

[B14] Carretié L. Hinojosa J. A. Martín-Loeches M. Mercado F. Tapia M. (2004). Automatic attention to emotional stimuli: neural correlates. Hum. Brain Mapp. 22, 290–299. 10.1002/hbm.20037 15202107 PMC6871850

[B15] Cespón J. Galdo-Álvarez S. Díaz F. (2012). The Simon effect modulates N2cc and LRP but not the N2pc component. Int. J. Psychophysiol. 84, 120–129. 10.1016/j.ijpsycho.2012.01.019 22326596

[B16] Codispoti M. De Cesarei A. Ferrari V. (2011). The influence of color on emotional perception of natural scenes. Psychophysiology 49, 11–16. 10.1111/j.1469-8986.2011.01284.x 22092301

[B17] Cousineau D. (2005). Confidence intervals in within-subject designs: a simpler solution to Loftus and Masson’ s method. Tutor. Quant. Methods Psychol. 1, 42–45.

[B18] Dehaene S. Bossini S. Giraux P. (1993). The mental representation of parity and number magnitude. J. Exp. Psychol. Gen. 122, 371–396 10.1037//0096-3445.122.3.371

[B19] Dekowska M. Kuniecki M. Jaśkowski P. (2008). Facing facts: neuronal mechanisms of face perception. Acta Neurobiol. Exp. (Wars) 68, 229–252. 18511959 10.55782/ane-2008-1692

[B20] Delplanque S. Lavoie M. E. Hot P. Silvert L. Sequeira H. (2004). Modulation of cognitive processing by emotional valence studied through event-related potentials in humans. Neurosci. Lett. 356, 1–4. 10.1016/j.neulet.2003.10.014 14746887

[B21] Delplanque S. N’diaye K. Scherer K. Grandjean D. (2007). Spatial frequencies or emotional effects?. A systematic measure of spatial frequencies for IAPS pictures by a discrete wavelet analysis. J. Neurosci. Methods 165, 144–150. 10.1016/j.jneumeth.2007.05.030 17629569

[B22] Eimer M. Kiss M. (2007). Attentional capture by task-irrelevant fearful faces is revealed by the N2pc component. Biol. Psychol. 74, 108–112. 10.1016/j.biopsycho.2006.06.008 16899334 PMC2375010

[B23] Eimer M. van Velzen J. (2006). Covert manual response preparation triggers attentional modulations of visual but not auditory processing. Clin. Neurophysiol. 117, 1063–1074. 10.1016/j.clinph.2006.01.005 16516545

[B24] Eimer M. van Velzen J. Driver J. (2002). Cross-modal interactions between audition, touch and vision in endogenous spatial attention: ERP evidence on preparatory states and sensory modulations. J. Cogn. Neurosci. 14, 254–271. 10.1162/089892902317236885 11970790

[B25] Eimer M. van Velzen J. Forster B. Driver J. (2003). Shifts of attention in light and in darkness: an ERP study of supramodal attentional control and crossmodal links in spatial attention. Brain Res. Cogn. Brain Res. 15, 308–323. 10.1016/s0926-6410(02)00203-3 12527104

[B26] Elliot A. J. Aarts H. (2011). Perception of the color red enhances the force and velocity of motor output. Emotion 11, 445–449. 10.1037/a0022599 21500913

[B27] Elliot A. J. Kayser D. N. Greitemeyer T. Lichtenfeld S. Gramzow R. H. Maier M. A.. (2010). Red, rank and romance in women viewing men. J. Exp. Psychol. Gen. 139, 399–417. 10.1037/a0019689 20677892

[B28] Elliot A. J. Maier M. A. (2012). Color-in-context theory. Adv. Exp. Soc. Psychol. 45, 61–125 10.1016/b978-0-12-394286-9.00002-0

[B29] Elliot A. J. Pazda A. D. (2012). Dressed for sex: red as a female sexual signal in humans. PLoS One 7:e34607. 10.1371/journal.pone.0034607 22514643 PMC3326027

[B30] Feldmann-Wüstefeld T. Schmidt-Daffy M. Schubö A. (2011). Neural evidence for the threat detection advantage: differential attention allocation to angry and happy faces. Psychophysiology 48, 697–707. 10.1111/j.1469-8986.2010.01130.x 20883506

[B31] Feng C. Li W. Tian T. Luo Y. Gu R. Zhou C.. (2014). Arousal modulates valence effects on both early and late stages of affective picture processing in a passive viewing task. Soc. Neurosci. 9, 364–377. 10.1080/17470919.2014.896827 24601745

[B32] Fenker D. B. Heipertz D. Boehler C. N. Schoenfeld M. A. Noesselt T. Heinze H.-J.. (2010). Mandatory processing of irrelevant fearful face features in visual search. J. Cogn. Neurosci. 22, 2926–2938. 10.1162/jocn.2009.21340 19702468

[B33] Forster K. I. Forster J. C. (2003). DMDX: a windows display program with millisecond accuracy. Behav. Res. Methods Instrum. Comput. 35, 116–124. 10.3758/bf03195503 12723786

[B34] Fortier-Gauthier U. Dell’acqua R. Jolicoeur P. (2013). The “red-alert” effect in visual search: evidence from human electrophysiology. Psychophysiology 50, 671–679. 10.1111/psyp.12050 23577877

[B35] Foxe J. J. Simpson G. V. (2002). Flow of activation from V1 to frontal cortex in humans. A framework for defining “early” visual processing. Exp. Brain Res. 142, 139–150. 10.1007/s00221-001-0906-7 11797091

[B36] Gegenfurtner K. R. (2003). Cortical mechanisms of colour vision. Nat. Rev. Neurosci. 4, 563–572. 10.1038/nrn1138 12838331

[B37] Gegenfurtner K. R. Rieger J. (2000). Sensory and cognitive contributions of color to the recognition of natural scenes. Curr. Biol. 10, 805–808. 10.1016/s0960-9822(00)00563-7 10898985

[B38] Gherri E. van Velzen J. Eimer M. (2007). Dissociating effector and movement direction selection during the preparation of manual reaching movements: evidence from lateralized ERP components. Clin. Neurophysiol. 118, 2031–2049. 10.1016/j.clinph.2007.06.003 17646131 PMC2386665

[B39] Hagemann N. Strauss B. Leissing J. (2008). When the referee sees red… Psychol. Sci. 19, 769–771. 10.1111/j.1467-9280.2008.02155.x 18816283

[B40] Hansen T. Gegenfurtner K. R. (2009). Independence of color and luminance edges in natural scenes. Vis. Neurosci. 26, 35–49. 10.1017/s0952523808080796 19152717

[B41] Harter M. R. Miller S. L. Price N. J. LaLonde M. E. Keyes A. L. (1989). Neural processes involved in directing attention. J. Cogn. Neurosci. 1, 223–237. 10.1162/jocn.1989.1.3.223 23968506

[B42] Hill R. A. Barton R. A. (2005). Psychology: red enhances human performance in contests. Nature 435:293. 10.1038/435293a 15902246

[B43] Hopf J. M. Mangun G. R. (2000). Shifting visual attention in space: an electrophysiological analysis using high spatial resolution mapping. Clin. Neurophysiol. 111, 1241–1257. 10.1016/s1388-2457(00)00313-8 10880800

[B44] Hubbard E. M. Piazza M. Pinel P. Dehaene S. (2005). Interactions between number and space in parietal cortex. Nat. Rev. Neurosci. 6, 435–448. 10.1038/nrn1684 15928716

[B45] Humphrey N. (1976). “The colour currency of nature,” in Colour for Architecture, ed Mikellides B. (London: Macmillan), 95–98.

[B46] Hutchings J. B. (1997). “Color in plants, animals and man,” in Color for Science, Art and Technology, ed Nassau K. (Amsterdam: Elsevier), 222–246.

[B47] Ikeda K. Sugiura A. Hasegawa T. (2013). Fearful faces grab attention in the absence of late affective cortical responses. Psychophysiology 50, 60–69. 10.1111/j.1469-8986.2012.01478.x 23153284

[B48] Jongen E. M. M. Smulders F. T. Y. Van der Heiden J. S. H. (2007). Lateralized ERP components related to spatial orienting: discriminating the direction of attention from processing sensory aspects of the cue. Psychophysiology 44, 968–986. 10.1111/j.1469-8986.2007.00557.x 17617171

[B49] Junghöfer M. Bradley M. M. Elbert T. R. Lang P. J. (2001). Fleeting images: a new look at early emotion discrimination. Psychophysiology 38, 175–178. 10.1111/1469-8986.3820175 11347862

[B50] Kayser D. N. Elliot A. J. Feltman R. (2010). Red and romantic behavior in men viewing women. Eur. J. Soc. Psychol. 40, 901–908 10.1002/ejsp.757

[B51] Keil A. Stolarova M. Moratti S. Ray W. J. (2007). Adaptation in human visual cortex as a mechanism for rapid discrimination of aversive stimuli. Neuroimage 36, 472–479. 10.1016/j.neuroimage.2007.02.048 17451974 PMC2034335

[B52] Kim M. S. Cave K. R. (1999). Top-down and bottom-up attentional control: on the nature of interference from a salient distractor. Percept. Psychophys. 61, 1009–1023. 10.3758/bf03207609 10497423

[B53] King T. (2005). Human color perception, cognition and culture: why “Red” is always red. Proc. SPIE Int. Soc. Opt. Eng. 5667, 234–242 10.1117/12.597146

[B54] Kuniecki M. Urbanik A. Sobiecka B. Kozub J. Binder M. (2003). Central control of heart rate changes during visual affective processing as revealed by fMRI. Acta Neurobiol. Exp. (Wars) 63, 39–48. 12784931 10.55782/ane-2003-1453

[B55] Lang P. J. Bradley M. M. Cuthbert B. N. (1998a). Emotion, motivation and anxiety: brain mechanisms and psychophysiology. Biol. Psychiatry 44, 1248–1263. 10.1016/s0006-3223(98)00275-3 9861468

[B56] Lang P. J. Bradley M. M. Cuthbert B. N. (1999). International Affective Picture System (IAPS): Instruction Manual and Affective Ratings. Technical Report A-4, Gainesville, Florida: The Center for Research in Psychophysiology, University of Florida.

[B57] Lang P. J. Bradley M. M. Fitzsimmons J. R. Cuthbert B. N. Scott J. D. Moulder B.. (1998b). Emotional arousal and activation of the visual cortex: an fMRI analysis. Psychophysiology 35, 199–210. 10.1111/1469-8986.3520199 9529946

[B58] Li F. F. VanRullen R. Koch C. Perona P. (2002). Rapid natural scene categorization in the near absence of attention. Proc. Natl. Acad. Sci. U S A 99, 9596–9601. 10.1073/pnas.092277599 12077298 PMC123186

[B59] Luck S. J. (2014). An Introduction to the Event-Related Potential Technique. Cambridge, MA: MIT, Chapter 11.

[B60] Luck S. J. Hillyard S. A. (1994). Electrophysiological correlates of feature analysis during visual search. Psychophysiology 31, 291–308. 10.1111/j.1469-8986.1994.tb02218.x 8008793

[B61] MacLeod C. Mathews A. Tata P. (1986). Attentional bias in emotional disorders. J. Abnorm. Psychol. 95, 15–20. 10.1037/0021-843x.95.1.15 3700842

[B62] Maier M. A. Barchfeld P. Elliot A. J. Pekrun R. (2009). Context specificity of implicit preferences: the case of human preference for red. Emotion 9, 734–738. 10.1037/a0016818 19803595

[B63] Milinski M. Bakker T. C. M. (1990). Female sticklebacks use male coloration in mate choice and hence avoid parasitized males. Nature 344, 330–333 10.1038/344330a0

[B64] Moller A. C. Elliot A. J. Maier M. A. (2009). Basic hue-meaning associations. Emotion 9, 898–902. 10.1037/a0017811 20001133

[B65] Nobre A. C. Sebestyen G. N. Miniussi C. (2000). The dynamics of shifting visuospatial attention revealed by event-related potentials. Neuropsychologia 38, 964–974. 10.1016/s0028-3932(00)00015-4 10775707

[B66] Öhman A. Lundqvist D. Esteves F. (2001). The face in the crowd revisited: a threat advantage with schematic stimuli. J. Pers. Soc. Psychol. 80, 381–396. 10.1037//0022-3514.80.3.381 11300573

[B67] Olofsson J. K. Nordin S. Sequeira H. Polich J. (2008). Affective picture processing: an integrative review of ERP findings. Biol. Psychol. 77, 247–265. 10.1016/j.biopsycho.2007.11.006 18164800 PMC2443061

[B68] Osorio D. Vorobyev M. (2005). Photoreceptor spectral sensitivities in terrestrial animals: adaptations for luminance and colour vision. Proc. Biol. Sci. 272, 1745–1752. 10.1098/rspb.2005.3156 16096084 PMC1559864

[B69] Palmer S. E. Schloss K. B. (2010). An ecological valence theory of human color preference. Proc. Natl. Acad. Sci. U S A 107, 8877–8882. 10.1073/pnas.0906172107 20421475 PMC2889342

[B70] Payen V. Elliot A. J. Coombes S. A. Chalabaev A. Brisswalter J. Cury F. (2011). Viewing red prior to a strength test inhibits motor output. Neurosci. Lett. 495, 44–48. 10.1016/j.neulet.2011.03.032 21406218

[B71] Peelen M. V. Fei-Fei L. Kastner S. (2009). Neural mechanisms of rapid natural scene categorization in human visual cortex. Nature 460, 94–97. 10.1038/nature08103 19506558 PMC2752739

[B72] Phelps E. A. Ling S. Carrasco M. (2006). Emotion facilitates perception and potentiates the perceptual benefits of attention. Psychol. Sci. 17, 292–299. 10.1111/j.1467-9280.2006.01701.x 16623685 PMC1555625

[B73] Posner M. I. Snyder C. R. Davidson B. J. (1980). Attention and the detection of signals. J. Exp. Psychol. 109, 160–174. 10.1037/0096-3445.109.2.160 7381367

[B74] Pourtois G. Schettino A. Vuilleumier P. (2013). Brain mechanisms for emotional influences on perception and attention: what is magic and what is not. Biol. Psychol. 92, 492–512. 10.1016/j.biopsycho.2012.02.007 22373657

[B75] Praamstra P. Boutsen L. Humphreys G. W. (2005). Frontoparietal control of spatial attention and motor intention in human EEG. J. Neurophysiol. 94, 764–774. 10.1152/jn.01052.2004 15744008

[B76] Praamstra P. Kourtis D. (2010). An early parietal ERP component of the frontoparietal system: EDAN not = N2pc. Brain Res. 1317, 203–210. 10.1016/j.brainres.2009.12.090 20059986

[B77] Praamstra P. Kourtis D. Nazarpour K. (2009). Simultaneous preparation of multiple potential movements: opposing effects of spatial proximity mediated by premotor and parietal cortex. J. Neurophysiol. 102, 2084–2095. 10.1152/jn.00413.2009 19657085 PMC6007848

[B78] Praamstra P. Oostenveld R. (2003). Attention and movement-related motor cortex activation: a high-density EEG study of spatial stimulus-response compatibility. Brain Res. Cogn. Brain Res. 16, 309–322. 10.1016/s0926-6410(02)00286-0 12706212

[B79] Ranzini M. Dehaene S. Piazza M. Hubbard E. M. (2009). Neural mechanisms of attentional shifts due to irrelevant spatial and numerical cues. Neuropsychologia 47, 2615–2624. 10.1016/j.neuropsychologia.2009.05.011 19465038

[B80] Rousselet G. Joubert O. Fabre-Thorpe M. (2005). How long to get to the “gist” of real-world natural scenes? Vis. Cogn. 12, 852–877 10.1080/13506280444000553

[B81] Schupp H. T. Junghöfer M. Weike A. I. Hamm A. O. (2004). The selective processing of briefly presented affective pictures: an ERP analysis. Psychophysiology 41, 441–449. 10.1111/j.1469-8986.2004.00174.x 15102130

[B82] Seiss E. Driver J. Eimer M. (2009). Effects of attentional filtering demands on preparatory ERPs elicited in a spatial cueing task. Clin. Neurophysiol. 120, 1087–1095. 10.1016/j.clinph.2009.03.016 19410504

[B83] Seiss E. Gherri E. Eardley A. F. Eimer M. (2007). Do ERP components triggered during attentional orienting represent supramodal attentional control? Psychophysiology 44, 987–990. 10.1111/j.1469-8986.2007.00591.x 17850244 PMC2248219

[B84] Sheliga B. M. Craighero L. Riggio L. Rizzolatti G. (1997). Effects of spatial attention on directional manual and ocular responses. Exp. Brain Res. 114, 339–351. 10.1007/pl00005642 9166923

[B85] Shepherd M. Müller H. J. (1989). Movement versus focusing of visual attention. Percept. Psychophys. 46, 146–154. 10.3758/bf03204974 2762102

[B86] Smith N. K. Cacioppo J. T. Larsen J. T. Chartrand T. L. (2003). May I have your attention, please: electrocortical responses to positive and negative stimuli. Neuropsychologia 41, 171–183. 10.1016/s0028-3932(02)00147-1 12459215

[B87] Sumner P. Mollon J. D. (2000). Catarrhine photopigments are optimized for detecting targets against a foliage background. J. Exp. Biol. 203, 1963–1986. 10851115 10.1242/jeb.203.13.1963

[B88] Surridge A. K. Osorio D. Mundy N. I. (2003). Evolution and selection of trichromatic vision in primates. Trends Ecol. Evol. 18, 198–205 10.1016/s0169-5347(03)00012-0

[B89] Talsma D. Slagter H. A. Nieuwenhuis S. Hage J. Kok A. (2005). The orienting of visuospatial attention: an event-related brain potential study. Brain Res. Cogn. Brain Res. 25, 117–129. 10.1016/j.cogbrainres.2005.04.013 15925498

[B90] Theeuwes J. (1992). Perceptual selectivity for color and form. Percept. Psychophys. 51, 599–606. 10.3758/bf03211656 1620571

[B91] Theeuwes J. Reimann B. Mortier K. (2006). Visual search for featural singletons: no top-down modulation, only bottom-up priming. Vis. Cogn. 14, 466–489 10.1080/13506280500195110

[B92] Thorpe S. Fize D. Marlot C. (1996). Speed of processing in the human visual system. Nature 381, 520–522. 10.1038/381520a0 8632824

[B93] van der Lubbe R. H. J. Neggers S. F. W. Verleger R. Kenemans J. L. (2006). Spatiotemporal overlap between brain activation related to saccade preparation and attentional orienting. Brain Res. 1072, 133–152. 10.1016/j.brainres.2005.11.087 16427618

[B94] Van der Lubbe R. Utzerath C. (2013). Lateralized power spectra of the EEG as an index of visuospatial attention. Adv. Cogn. Psychol. 9, 184–201. 10.2478/v10053-008-0144-7 24605177 PMC3902831

[B95] Van Strien J. W. Langeslag S. J. Strekalova N. J. Gootjes L. Franken I. H. (2009). Valence interacts with the early ERP old/new effect and arousal with the sustained ERP old/new effect for affective pictures. Brain Res. 1251, 223–235. 10.1016/j.brainres.2008.11.027 19063866

[B96] van Velzen J. Eimer M. (2003). Early posterior ERP components do not reflect the control of attentional shifts toward expected peripheral events. Psychophysiology 40, 827–831. 10.1111/1469-8986.00083 14696736

[B97] Waitt C. Gerald M. S. Little A. C. Kraiselburd E. (2006). Selective attention toward female secondary sexual color in male rhesus macaques. Am. J. Primatol. 68, 738–744. 10.1002/ajp.20264 16786524

[B98] Yiend J. (2010). The effects of emotion on attention: a review of attentional processing of emotional information. Cogn. Emot. 24, 3–47 10.1080/02699930903205698

